# Vitreociliary Block in a Patient With Uveitis and Previous Laser Posterior Capsulotomy

**DOI:** 10.7759/cureus.14786

**Published:** 2021-05-01

**Authors:** Ana-María Dorado-López-Rosado, Enrique Mencía-Gutiérrez, María Polo-García, Esperanza Gutiérrez-Díaz

**Affiliations:** 1 Ophthalmology, 12 de Octubre Hospital, Complutense University, Madrid, ESP; 2 Ophthalmology, Nuestra Señora de Sonsoles Hospital, Ávila, ESP

**Keywords:** vitreociliary block, aqueous misdirection syndrome, malignant glaucoma, laser capsulotomy, retinal vasoproliferative tumor

## Abstract

Purpose: To report a case of vitreociliary block (VCB) six months after a laser posterior capsulotomy (LPC). Case report: A 25-year-old man with uveitis, retinal vasoproliferative tumor, cataract, and acute angle-closure glaucoma due to pupillary seclusion, which required laser iridotomies, implantation of an Ahmed valve, phacoemulsification, and LPC. Six months after capsulotomy, he presented a generalized flattening of the anterior chamber (AC) and ocular hypertension, with patent iridotomies. Hyperechoic anterior hyaloid and hypoechoic spaces in the vitreous were seen in ultrasound imaging. The VCB did not respond to pharmacological treatment and was solved immediately after laser hyaloidotomy. Conclusion: There are three cases of VCB after LPC described in the literature. Our patient presented a chronic inflammatory process that generated an inflammatory membrane at the level of the anterior hyaloid with adhesion to the ciliary processes, causing posterior misdirection of the aqueous humor, decreased permeability of the anterior hyaloid, and finally, VCB.

## Introduction

Vitreociliary block (VCB) is characterized by generalized flattening of the anterior chamber (AC) associated with increased intraocular pressure (IOP) in the absence of pupillary block [1-3]. It was initially called malignant glaucoma by Albrecht von Graefe in 1869 due to its progressive course and its therapeutic complexity [1]. It has also been described as ciliary block glaucoma, VCB glaucoma, or aqueous humor misdirection syndrome, among others [4]. Although its etiology is not well known, several mechanisms have been proposed, and it is believed that it is the result of the passage of the aqueous humor in the posterior direction toward the vitreous, where it is trapped, together with poor conductivity of the aqueous humor through the vitreous and a decrease in the permeability of the anterior hyaloid, causing a pressure difference in the two compartments, and ultimately an anterior displacement of the iridocrystalline diaphragm, with flattening of the AC and secondary angle-closure glaucoma [3-5]. It usually occurs after ocular surgery in predisposed eyes; however, it has been described after laser iridotomy, laser posterior capsulotomy (LPC), laser cyclophotocoagulation, with the use of miotics, and it may even occur spontaneously in eyes not previously operated on [6].

The purpose of this article is to describe a case of VCB six months after a posterior laser Nd:YAG capsulotomy in a young patient with a history of intermediate uveitis and a vasoproliferative retinal tumor that was solved immediately after a laser hyaloidotomy was performed.

## Case presentation

A 25-year-old male with bilateral intermediate uveitis secondary to a vasoproliferative retinal tumor, and posterior development of cataract and acute glaucoma due to pupillary seclusion in both eyes. After two Nd:YAG laser iridotomies, the IOP remained high despite maximum medical treatment, and an Ahmed glaucoma valve was placed in both eyes, followed by phacoemulsification with intraocular lens implantation in the capsular bag in both eyes. Postoperative best-corrected visual acuity (BCVA) was 20/32 (Snellen scale) in the right eye (RE) and 20/40 in the left eye (LE). A month later, the LE required a pars plana vitrectomy and silicone tamponade due to retinal detachment, resulting in amaurosis.

**Figure 1:**
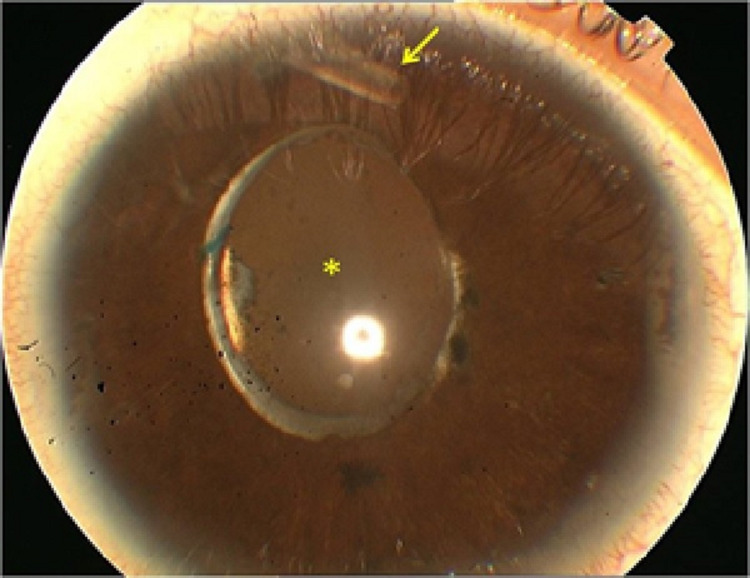
Slit-lamp photograph of the anterior segment of the patient's right eye at the time of vitreociliary blockage (VCB) where the end of the Ahmed valve tube correctly placed in the anterior chamber can be visualized (arrow) as well as the turbidity of the anterior hyaloid behind the intraocular lens (asterisk).

**Figure 2:**
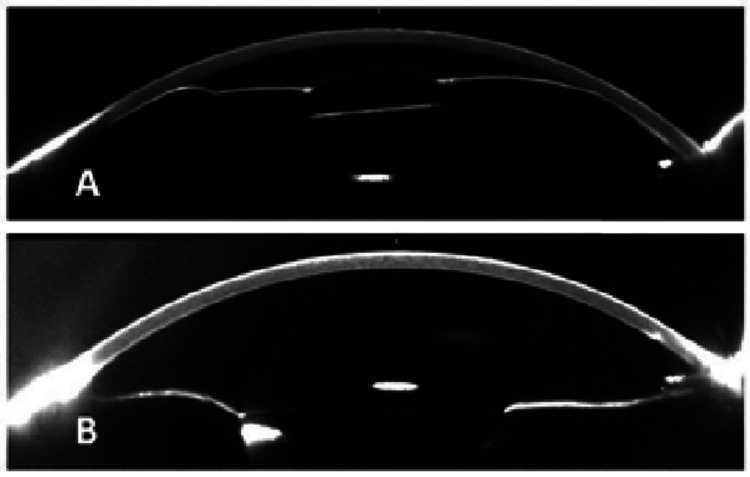
Scheimpflug image of the anterior segment of the patient with the VCB prior to performing hyaloidotomy with the Nd:YAG laser (A) and immediately after this procedure (B). VCB: Vitreociliary block.

**Figure 3:**
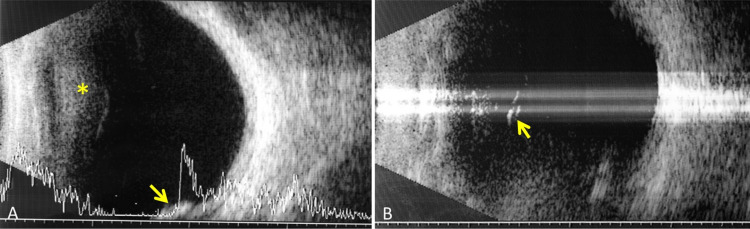
Longitudinal ultrasound in B-mode with associated A-mode of the right eye with the VCB, showing increased reflectivity behind the lens (asterisk) suggestive of inflammatory activity, empty spaces in the anterior vitreous compatible with aqueous humor collections, and an image of a nodular chorioretinal mass, of medium-high reflectivity, located inferiorly, corresponding to the vasoproliferative retinal tumor (arrow) (A) and artifacted B-mode ultrasound showing the retrolental inflammatory membrane (arrow) and magnification reflectivity in the vitreous gel (B). VCB: Vitreociliary block.

**Figure 4:**
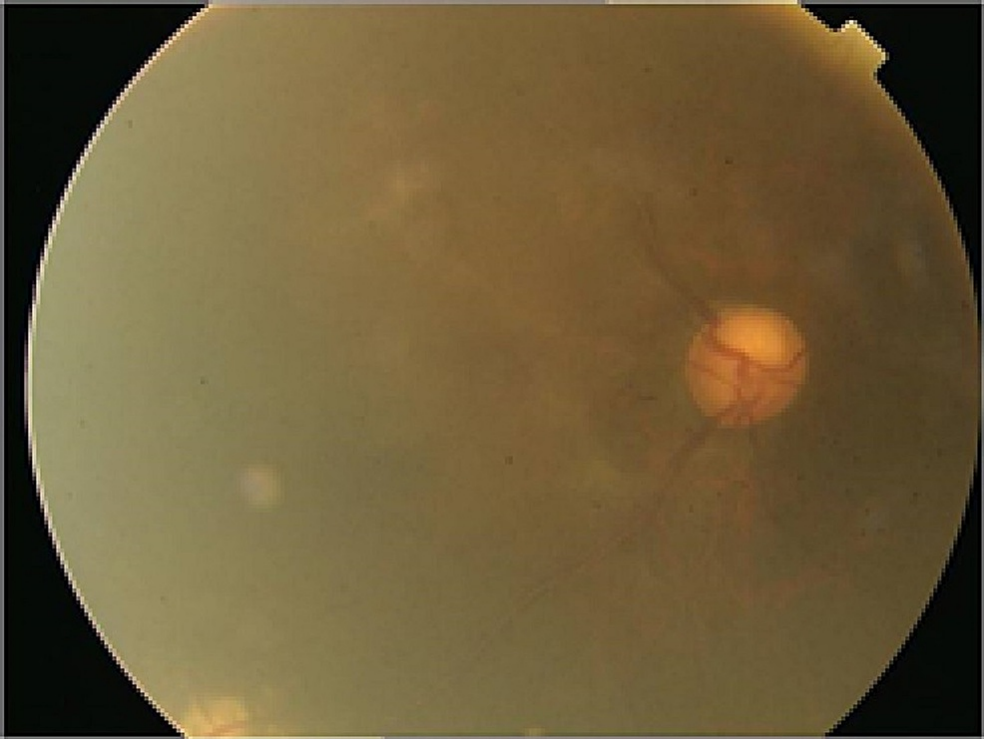
Retinography of the patient's right eye at the time of the VCB showing turbidity of the thickened anterior hyaloid and the anterior vitreous. Glaucomatous excavation is observed in the optic disc. VCB: Vitreociliary block.

Twenty months after the cataract surgery, a posterior capsulotomy with Nd:YAG laser was performed without complications in the RE due to capsular opacification, with the recovery of the previous BCVA of 20/32. Two months later, the patient developed repeated episodes of inflammation in AC with the closure of the iridotomies and anterior displacement of the iris, together with increased IOP in the RE. These episodes were satisfactorily managed with reopening of the iridotomies and topical corticosteroid therapy, and after them, the iridotomies remained patent, with open AC angle and normal IOP without hypotensive treatment. Six months after the capsulotomy, the patient presented with blurred vision. BCVA was 20/200, and a noticeable anterior displacement of the iridocrystalline diaphragm with generalized AC flattening was observed in the slit lamp. The iridotomies were patent but the IOP was 44 mmHg. The Ahmed valve maintained a good filtering bleb and the tube was correctly positioned in the AC (Figure 1). Behind the intraocular lens, an inflammatory membrane was observed with thickening of the anterior hyaloids, vitreous turbidity, and no signs of uveal effusion in the fundus. The depth and volume of AC estimated by OCULUS Pentacam® HR Type 70900, OCULUS Optikgeräte GmbH, Wetzlar, Germany, were greatly diminished, being 1.82 mm and 46 mm3, respectively (Figure 2A). Ocular ultrasound revealed a hyperechoic anterior hyaloid membrane, an increase in vitreous echogenicity with an empty space in the anterior vitreous compatible with aqueous humor collection; choroidal effusion was ruled out. An image of a nodular chorioretinal mass, located inferiorly, corresponding to the vasoproliferative retinal tumor was also found (Figure 3A). An inflammatory membrane was observed at the level of the anterior hyaloid and anterior vitreous turbidity (Figure 3B), which made it difficult to see the fundus (Figure 4). Although the iridotomies appeared patent, new peripheral iridotomies with Nd:YAG laser were performed without clinical improvement. Suspecting VCB, medical treatment was started with topical atropine, cycloplegic and phenylephrine, topical beta blockers and alpha agonists, oral acetazolamide, and intravenous mannitol. A total of 24 hours later, there was no improvement; therefore, an anterior hyaloidectomy with Nd:YAG laser was performed. A posterior displacement of the iridocrystalline diaphragm and an increase of the AC depth were immediately observed and the IOP dropped to 13 mmHg. The AC study using Pentacam® HR showed a depth of 3.85 mm and a volume of 235 mm3 (Figure 2B). After one year of follow-up, the patient maintains a BCVA of 20/32 and there has been no recurrence of the VCB in the RE.

## Discussion

It is estimated that VCB occurs in 2-4% of cataract-operated patients, more frequently after extracapsular surgery and after glaucoma surgery in eyes with previous chronic angle-closure glaucoma [4-6], and it is postulated to be caused by an increased postoperative inflammation [7]. Inflammation seems to play a role in the etiopathogenesis of VCB [7], as is the case reported in this article, in which there was a chronic underlying inflammatory process, which worsened after LPC, causing repeated iridotomy closures and acute glaucoma due to anterior displacement of the iris and VCB. In the slit-lamp examination at the time of the VCB, an inflammatory membrane was observed at the level of the anterior hyaloid, and anterior vitreous turbidity made it difficult to see the fundus. Ocular ultrasound revealed inflammatory activity in the anterior vitreous, a retrolental membrane that seemed to correspond to the anterior hyaloids and lagoons of low reflectivity in the anterior vitreous that suggested collections of aqueous humor. The performance of an ultrasonic biomicroscopy could have helped to identify the situation of the ciliary processes.

We think that in this patient the VCB was most likely triggered by the adhesion of an inflammatory membrane to the ciliary processes that directed the aqueous humor toward the vitreous cavity, where it remained trapped, together with the thickening of the anterior hyaloid as a result of inflammation, which decreased its permeability and prevented the passage of aqueous humor toward the AC. Only three cases of VCB after LPC have been reported in the literature, and all presented shortly after the procedure [8-10].

The initial treatment of VCB is pharmacological with topical cycloplegics/mydriatics, aqueous suppressants, and hyperosmotic agents with the aim of reducing the production of aqueous humor, dehydrating the vitreous, and displacing the iridocrystalline diaphragm backward [2]; however, in many cases, as in our patient, it cannot solve the situation. If the VCB does not resolve, in pseudophakic patients, Nd:YAG laser hyaloid capsulotomy is recommended in an attempt to relieve the blockage and reverse the aqueous misdirection. In refractory cases or phakic patients, a vitrectomy should be performed [4,6,7].

## Conclusions

In conclusion, VCB occurs as a result of multiple mechanisms involved simultaneously or sequentially. We present a case of VCB in a pseudophakic patient six months after an LPC was performed; he presented a chronic inflammatory process that favored its development, despite a previous capsulotomy. It is important to control the inflammation after LPC to avoid the appearance of this serious complication.
